# Ceftazidime–Avibactam Pharmacokinetic Comparative In Vivo/In Vitro Study in a Critically Ill Children Under High‐Volume Continuous Venovenous Hemodiafiltration

**DOI:** 10.1002/prp2.70139

**Published:** 2025-07-17

**Authors:** Michael Thy, Alexandre Debs, Gabrielle Lui, Leo Froelicher‐Bournaud, Jean‐Marc Tréluyer, Mehdi Oualha

**Affiliations:** ^1^ Service de Médecine Intensive et Réanimation Infectieuse Hôpital Bichat Claude‐Bernard, AP‐HP, Université Paris Cité Paris France; ^2^ UMR 1343, Evaluation Des thérapeutiques et Pharmacologie périnatale et pédiatrique INSERM, Université Paris Cité Paris France; ^3^ UMR 1137, IAME INSERM, Université Paris Cité Paris France; ^4^ Service de Réanimation et Surveillance Continue médicochirurgicales Hôpital Necker Enfants‐Malades, AP‐HP, Université Paris Cité Paris France; ^5^ Service de Pharmacologie Clinique Hôpital Cochin, AP‐HP, Université Paris Cité Paris France; ^6^ Unité de Recherche Clinique‐Centre D'investigation Clinique, CIC1419 Hôpital Cochin‐Necker, AP‐HP, Université Paris Cité Paris France

## Abstract

Ceftazidime–avibactam is a novel cephalosporin/beta‐lactamase inhibitor combination developed to address increasing antimicrobial resistance. This report presents a comparative study of the pharmacokinetics of ceftazidime and avibactam, utilizing in vitro data derived from two experiments with continuous venovenous hemodiafiltration (CVVHDF) simulation and a comparison with a previously published in vivo case report. The results highlight the importance of therapeutic drug monitoring and the need for higher dosing or continuous infusion of ceftazidime–avibactam in critically ill children under crontinuous renal replacement therapy (CRRT).

## 1 Introduction

Ceftazidime–avibactam is a novel cephalosporin/beta‐lactamase inhibitor combination developed to address the increasing prevalence of multidrug‐resistant Gram‐negative (MDR‐GN) pathogens [1, 2]. Its use in pediatric patients was approved by the US Food and Drug Administration in 2019 for intra‐abdominal infections, and recent literature reviews have demonstrated its efficacy and safety in various MDR‐GN infections [3, 4]. The pharmacokinetics (PK) of ceftazidime–avibactam under continuous renal replacement therapy (CRRT) have been described in a few adult cases, but data in pediatric patients are limited [5]. This study aims to describe the PK of ceftazidime–avibactam with in vitro data derived from two experiments with continuous veno‐venous hemodiafiltration (CVVHDF) simulation, then compare in vitro results with a previously published in vivo case report of a critically ill pediatric patient undergoing CRRT.

## 2 Methods

### 2.1 In Vivo Study

The in vivo data were obtained from a case report of a 6‐month‐old, 8‐kg‐weighted, female with sepsis due to *Stenotrophomonas maltophilia* infection following stem cell transplantation [5]. The patient was treated with ceftazidime–avibactam every 8 h with a 2‐h infusion of 30–7.5 mg/kg.

Residual diuresis during CVVHDF remained stable at approximately 0.5 mL/kg/h.

CVVHDF was performed using the Prismaflex system machine (Baxter Int, SE) with an ST 60 set. Phoxilium was used as both the dialysate and replacement solution. Anticoagulation was avoided due to hemorrhagic risk and liver failure. The blood flow rate was maintained at 80 mL/min (10 mL/kg/min), with a dialysate flow rate of 300 mL/h (37.5 mL/kg/h), a replacement flow rate of 600 mL/h (75 mL/kg/h), and a net ultrafiltrate flow rate ranging from 30 to 40 mL/h (3.75–5 mL/kg/h). This resulted in a total effluent rate of 930–940 mL/h (116.2–117.5 mL/kg/h), a critical parameter impacting drug clearance.

### 2.2 In Vitro Study

In vitro experiments were conducted to simulate the conditions of CRRT [6]. The experiments were performed using the Prismaflex system machine (Baxter Int, SE) with an ST 60 set. Dialysate and replacement solutes were Phoxilium. No anticoagulation was administered. The weight was set to 8 kg, the blood flow rate to 80 mL/min (10 mL/kg/min for an 8 kg patient); dialysate flow rate to 300 mL/h (37.5 mL/kg/h); replacement flow rate to 600 mL/h (75 mL/kg/h) and net ultrafiltrate flow rate to 35 mL/h (4.4 mL/kg/h). The total effluent rate was 935 mL/h (117.5 mL/kg/h).

The circuit was primed with 1 L of 0.9% NaCl containing heparin to prevent clotting. Ceftazidime–avibactam was both prepared at a concentration of 50 mg/L by adding 250 mg of each antibiotic to 5 L of Phoxylium.

An adsorption and steady state phase of 60 min was conducted by running the CVVHDF system with the only antibiotic solution circulating. After this initial phase, sample collections were collected at multiple time points (0 to 360 min) to assess drug concentration decay over time from four distinct sites: prefilter (E), postfilter (S), effluent (F), and the reservoir as a control (T). Each sample underwent analysis to measure the concentrations of ceftazidime–avibactam using validated high‐performance liquid chromatography techniques, allowing for accurate monitoring of pharmacokinetic changes over time.

## 3 Results

In vitro data for ceftazidime and avibactam are summarized in Table 1, showing the median and interquartile ranges for the concentrations at different time points.

**TABLE 1:** Median concentrations with interquartile range of Ceftazidime and Avibactam in each sample type under CVVHDF.

Compound name	Sample type	Median concentration (mg/L)	Interquartile range (mg/L)
Ceftazidime	T (Control)	42.6	42.4–42.9
	E (Prefilter)	37.2	31.2–41.4
	S (Postfilter)	31.8	27.0–39.6
	F (Effluent)	36.4	27.7–41.3
Avibactam	T (Control)	11.9	11.8–12.0
	E (Prefilter)	10.2	7.9–11.4
	S (Postfilter)	7.5	6.9–9.7
	F (Effluent)	9.2	6.6–10.6

**FIGURE 1:**
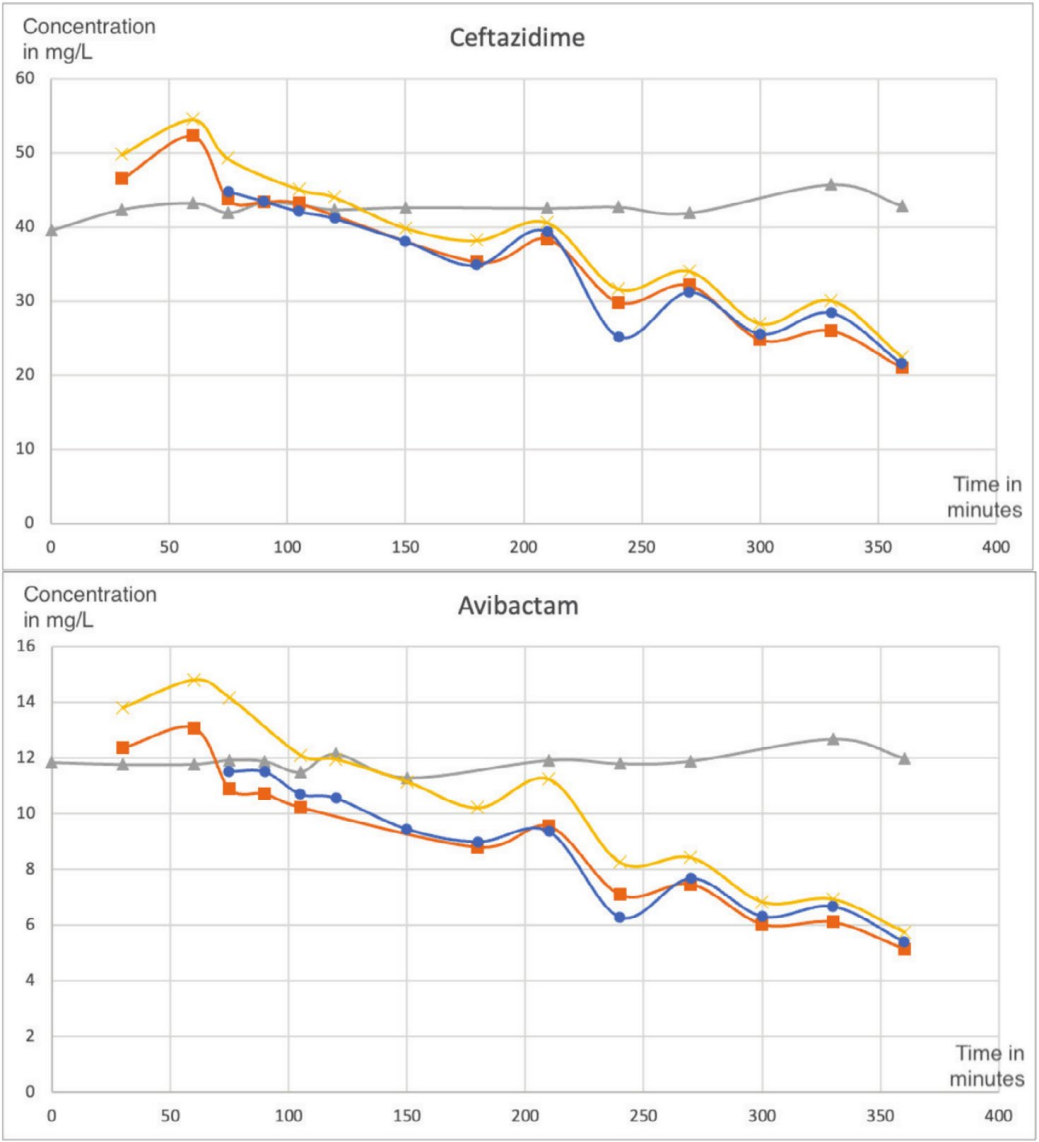
Concentration dynamics of ceftazidime and avibactam over time under CVVHDF. Gray line for control sample (T), yellow line for prefilter sample (E), orange line for postfilter sample (S), blue line for effluent sample (F).

Blood samples were collected at various time points to evaluate the pharmacokinetics of the administered drugs during CVVHDF. Timepoint 0 represents the baseline sample taken immediately before antibiotic administration. Timepoint 30 was collected 30 min after drug administration to assess the initial concentration and potential adsorption by the CVVHDF filter. Timepoint 60 was collected at 60 min before initiation of the CRRT by CVVHDF. Timepoints 75 to 360 were obtained at regular intervals (every 15 to 30 min) to capture both the distribution and elimination phases of the drug. This sampling strategy provided comprehensive monitoring of the drug's pharmacokinetics over time (Figure 1 and Table 1). A comparison with the in vivo case report is displayed in Table 2.

**TABLE 2:** Comparison of in vivo and in vitro clearances for ceftazidime and avibactam.

Parameter	In vivo (Ceftazidime)	In vitro (Ceftazidime)	In vivo (Avibactam)	In vitro (Avibactam)
CL (L/h)	1.7		3.02	
CLCRRT (L/h)	0.49–1.02	0.85 [0.82–0.86]	0.43–0.99	0.80 [0.76–0.83]

## 4 Discussion

The in vitro data provide valuable insights into the pharmacokinetics of ceftazidime–avibactam under CRRT conditions. The in vitro data showed a significant decrease in the concentrations of ceftazidime and avibactam over time, particularly in the prefilter and postfilter samples. This confirms that the drugs are being effectively removed by the CRRT system, which is consistent with the high clearance rates observed in the in vivo study. The effluent samples also showed a significant decrease in the concentrations of both drugs, indicating that the drugs are being eliminated through the CRRT system. The significant decrease in the concentrations of ceftazidime and avibactam over time suggests that higher dosing or continuous infusion may be necessary to maintain therapeutic levels and prevent the development of microbiological resistance. The in vitro data also highlight the importance of therapeutic drug monitoring in critically ill patients undergoing CRRT.

The in vitro clearances (CLCRRT) for both ceftazidime and avibactam were less variable than the corresponding in vivo CRRT clearances. This aligns with the hypothesis that the in vitro model corresponds to an ideal PK model due to the absence of complicating factors such as blood, albumin, and clotting, which can hinder filter efficiency in vivo.

In vivo total clearance (CL) is known to surpass CRRT clearance (CLCRRT) due to residual endogenous elimination pathways such as residual renal function. While in vitro models provide valuable experimental control and are instrumental in developing physiologically based pharmacokinetic (PBPK) models, they lack the contribution of these endogenous processes, emphasizing their limitations. Furthermore, in vitro models demonstrate lower variability compared to in vivo data, reflecting their reproducibility and precision. However, this reduced variability highlights the need to account for the more complex and variable conditions present in clinical settings when interpreting in vitro findings.

Thus, while in vitro models offer significant advantages for hypothesis testing and parameter estimation, they complement rather than replace in vivo studies, which remain essential for capturing the full spectrum of pharmacokinetic variability in patients [7].

## 5 Conclusion

The in vitro data provide valuable insights into the pharmacokinetics of ceftazidime–avibactam under CRRT conditions in critically ill children, highlighting the importance of therapeutic drug monitoring and the need for higher dosing or continuous infusion in this context. The study underscores the utility of in vitro experiments in predicting the clearance of drugs by CRRT.

## Author Contributions

M.T. and M.O. conceived the study and the in vitro model. M.T. and A.D. collected the data. L.F.‐B. and G.L. performed the dosages. J.‐M. T. contributed to the review and edit the final manuscript. M.T. and M.O. contributed to the acquisition, analysis, and interpretation of the results. M.T. and M.O. drafted the manuscript and critically revised the manuscript.

## Ethics Statement

The authors have nothing to report.

## Consent

All authors critically revised the manuscript and consent for publication.

## Conflicts of Interest

The authors declare no conflicts of interest.

## Data Availability

All data generated or analyzed during this study are included in this study or its Supporting Information files. Further inquiries can be directed to the corresponding author.
